# Construction of a N-CDs/AuNCs@ZIF-8-assisted ratiometric fluorescent nanosensor for glyphosate detection in edible and medicinal malt

**DOI:** 10.1016/j.fochx.2024.101983

**Published:** 2024-11-08

**Authors:** Doudou Lei, Lingling Li, Pengyue Song, QingBin Xu, Lihua Huang, Xiao Ma, Lidong Zhou, Weijun Kong

**Affiliations:** a State Key Laboratory of Southwestern Chinese Medicine Resources, Pharmacy College, Chengdu University of Traditional Chinese Medicine, Chengdu 611137, China; b Institute of Medicinal Plant Development, Chinese Academy of Medical Sciences and Peking Union Medical College, Beijing 100193, China; c School of Traditional Chinese Medicine, Capital Medical University, Beijing 100069, China

**Keywords:** Ratiometric fluorescent nanosensor, N-CDs/AuNCs@ZIF-8 dual-emissive probes, Ultrasensitive detection, Glyphosate, Edible and medicinal malt

## Abstract

Glyphosate (Gly) is a widely-used herbicide in food production, while, the residue of which due to the long-term and excessive overspray poses serious threats to public health. The development of reliable methods for its sensitive detection is of great urgency. In this study, a novel ratiometric fluorescent nanosensor by encapsulating N-doped carbon dots (N-CDs) and gold nanoclusters (AuNCs) in zeolitic imidazole framework-8 (ZIF-8) as the dual-emissive fluorescence probes at 410 and 650 nm, respectively, was constructed for Gly detection. Due to the adsorption property of ZIF-8, the N-CDs/AuNCs@ZIF-8 nanoprobes accumulated Cu^2+^ to quench the red fluorescence of AuNCs, and the blue fluorescence of N-CDs was stable. While thiocholine, a product of acetylthiocholine, hydrolyzed by acetylcholinesterase could coordinate with Cu^2+^, resulting in significant fluorescence recovery of AuNCs. This phenomenon was utilized for the quantitation of Gly, due to its inhibitory effect on acetylcholinesterase activity. By calculating the fluorescence intensity ratio (*I*_650_*/I*_410_), Gly in real sample could be accurately determined in a concentration range of 2–100 ng/mL with a limit of detection of 1.92 ng/mL because of the anti-interference and the self-correction ability of the two fluorescence signals. The N-CDs/AuNCs@ZIF-8 nanoprobes-assisted ratiometric fluorescent nanosensor exhibited unique merits of rapid construction, simple operation, high specificity, and good accuracy for Gly in edible and medicinal malt samples with recoveries of 93.7–108.2 %. This study presents a multiple tool for the versatile sensing of trace pesticides in more food matrices, which can be extended to a full range of environmental and food safety applications.

## Highlights

• A ratiometric fluorescent nanosensor was developed.
• N-CDs and AuNCs were encapsulated into ZIF-8 for constructing dual-emissive fluorescent probes.
• The “signal on-off-on-off” strategy was introduced for ratiometric sensing of glyphosate.
• A low LOD of 1.92 ng/mL for glyphosate was due to the anti-interference and self-correction ability.
• Real application in malt confirmed its practicability and great potential in complex food matrices.

## 1 Introduction

Glyphosate (N-(phosphonomethyl)glycine), abbreviated as Gly, is a systemic broad-spectrum organophosphate pesticide that has become the most widely used and best-selling herbicide. The annual global use of glyphosate has reached 825,800 tons, and more than 700 products containing glyphosate are used in around 130 countries (Nova, Calheiros, & Silva, 2020). The production and consumption of Gly herbicide has increased dramatically since the 20th century, accounting for more than three-quarters of all pesticide use (Singh et al., 2020). Widespread overspray of Gly leads to residue in the soil, water and the environment, and finally accumulation in the human body through the food chain (Xu, Smith, Smith, Wang, & Li, 2019; Yaah, Ahmadi, Morales-Torres, & Ojala, 2024). Several studies have confirmed that long-term exposure to Gly in humans causes damage to the central nervous system, leading to respiratory, cardiac and neuromuscular dysfunction, and reduces male fertility (Ma et al., 2023; Xiao, Wei, Yang, & Wang, 2023). Therefore, the development of an expedient and generalized means for Gly monitoring is of great importance and necessity.

Conventional methods, such as liquid/gas chromatography coupled with diverse detectors (Malla, Dubey, Kumar, Yadav, & Kumari, 2023; Pires, de Araújo, Oliveira-Filho, & Caldas, 2023; Wang et al., 2023), and ion chromatography (Amberger, Schröder, Kuballa, & Jantzen, 2023), have been applied for the detection of Gly. Although these methods are highly sensitive, they have the disadvantages of bulky equipment, complex sample pre-treatment, high cost, and harsh requirements for staff, which make it difficult to meet the demand for rapid, high-volume sample testing in the field (Rawat et al., 2023; Wei et al., 2024). As a preferred alternative, the fluorescent sensor has garnered increasing interest in the detection of trace pesticides due to its high selectivity, simplicity, real-time detection, and good reproducibility (Li, Pan, Wang, Wang, & Peng, 2024; Zhu, Lu, Xu, & Xiao, 2024). Compared to the single-emissive fluorescence detection mode that might be affected by the photobleaching effects to give false-positive results, the dual-probe based ratiometric fluorescent sensors could significantly improve analytical accuracy due to their dual-emissive signal self-correction ability to effectively eliminate the external environmental disturbances caused by single-emissive signal error bias (Deng et al., 2024; Ma et al., 2023; Tong et al., 2023).

Recently, various nanolabels have attracted much attention to constructing the ratiometric fluorescent signal probes with one fluorophore acting as a reference probe and the other as a signal probe (Chen et al., 2024). Of them, nitrogen-doped carbon dots (N-CDs), as zero-dimensional nanoluminescent materials with green synthesis, rich functional groups, suitable optical characteristics, good environmental friendliness, and high photostability have obtained worldwide attention to replacing conventional fluorophores and specific applications in constructing diverse sensing platform in the biomedicine, environmental monitoring, and food safety fields (Fu, Li, Lu, Dong, & Yang, 2024; Zhang et al., 2022). In addition, gold nanoclusters (AuNCs) have also been popularized as promising fluorescent nanolabels in sensing applications in merits of their tunable emission, large Stokes shift, high fluorescence intensity, good compatibility with biomolecules and low toxicity (Farkhani et al., 2024; Zhang et al., 2024). Benefiting from their good photoluminescence properties, N-CDs and AuNCs have been regarded as perfect substitutes for traditional fluorophores to fabricate the ratiometric fluorescence nanoprobes by simple mixing reactions, but they exhibited low and easily-interfered fluorescence responses. Thus, further exploring a reliable strategy to enhance the luminescence efficiency of N-CDs and AuNCs to improve the detection sensitivity is necessary.

Metal-organic frameworks (MOFs), the complex porous materials composed of inorganic clusters and organic ligands with large specific surface area, good crystalline structure, adjustable pore size and abundant functional groups, have been used to encapsulate a large number of fluorescence probes (Wu et al., 2023) to greatly improve the luminescence efficiency of the loaded fluorescent probes for amplifying the sensing signals (Mansour et al., 2024; Saeed et al., 2024). As a prime example, MOFs were used to coat various fluorescence probes to develop diverse sensors for sensitive detection of different targets (Chen et al., 2024; Yang et al., 2024; Zhang et al., 2023; Zhang et al., 2024). Of them, ZIF-8 MOF has got more attention in constructing different sensing platforms. For example, Karimzadeh, Gharekhani, Rahimpour, and Jouyben (2023) successfully synthesized N-CQDs@UiO-66/PVA nanocomposites for ratiometric fluorescent detection of pethidine. Wei et al. (2023) established a GSH-Au NCs@ZIF-8 hydrogel-based sensor for the field detection of copper ions and glyphosate.

**Scheme 1:**
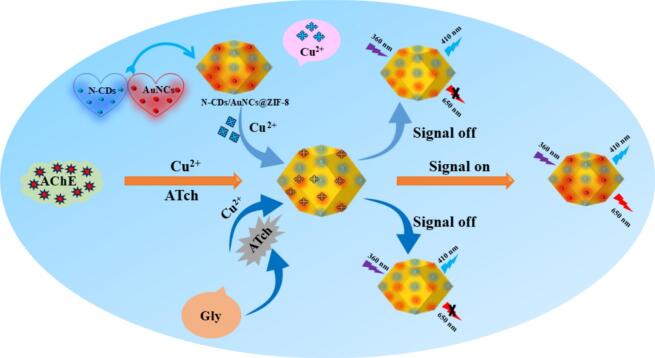
Illustration of the dual-emissive N-CDs/AuNCs@ZIF-8 nanoprobes assisted ratiometric fluorescent nanosensor for Gly detection.

Enlightened by the above analysis, a novel N-CDs/AuNCs@ZIF-8 dual-emissive nanoprobes based ratiometric fluorescent was developed for Gly detection (Scheme 1). The blue-emissive N-CDs were encapsulated into high-porosity ZIF-8 as reference probes and the encapsulated red-emissive AuNCs were regarded as signal labels. The prepared N-CDs/AuNCs@ZIF-8 nanoprobes showed two emission peaks at 410 and 650 nm under a single excitation wavelength of 360 nm. When copper ions exist (Cu^2+^), the red fluorescence of AuNCs was gradually quenched, while the blue fluorescence of N-CDs was maintained. Thiocholine (Tch), an outcome of acetylthiocholine (ATch) hydrolyzed by acetylcholinesterase (AChE), could couple to Cu^2+^ *via* the sulfhydryl (-SH) group, resulting in a marked red fluorescence restoration of AuNCs, which was quenched again because Gly could inhibit the AChE activity. By evaluating the changes in the fluorescence intensity ratio of N-CDs and AuNCs (*I*_650_*/I*_410_) after the addition of Gly, the N-CDs/AuNCs@ZIF-8 nanoprobes-assisted “signal on-off-on-off” ratiometric fluorescent nanosensor could achieve accurate determination of Gly in a range of 2–100 ng/mL. The viability of the developed ratiometric fluorescence sensor was confirmed in real edible and medicinal malt samples (Chen, 2023), suggesting that the sensor could be used for pesticide detection in other complex food matrices.

## 2 Experiments

### 2.1 Materials and reagents

Malt samples were collected from Hebei province (batch number 211201, Hebei, China). Chloroauric acid (HAuCl_4_) was purchased from Wuhan Ouellette Biotechnology Co., Ltd. (Wuhan, China). Zn(NO_3_)_2_-6H_2_O, 2-methylimidazole, urea (purity ≥99.0 %) and anhydrous citric acid (purity ≥99.5 %) were bought from Aladdin Biochemistry and Technology (Shanghai, China). Bovine serum albumin (BSA), acetylthiocholine iodide (ATch), acetylcholinesterase AChE, and glyphosate (Gly) standard solution were obtained from Beijing Inokai Technology Co., Ltd. (Beijing, China). Pesticide standards of thiram, carbendazim atrazine and metribuzin were provided by Shanghai Amperexperiment Technology Co., Ltd. (Shanghai, China).

### 2.2 Apparatus

Material characterization was done on an FEI Talos F200X (FEI, Tecnai, USA) transmission electron microscope (TEM), a high-resolution transmission electron microscope (HR-TEM) and a scanning electron microscope (SEM) Microscope: Hitachi SU 8020, (Japan Energy Spectroscopy: HORIBA EX250, Japan Horiba HORIBA Origin, Japan). Fourier transform infrared (FT-IR) spectra were measured by an iS10 FT-IR spectrometer (Nicorette, USA). X-ray powder diffraction (XRD) patterns were recorded on an X-ray powder diffractometer D8 ADVANCE (Bruker, Germany).

### 2.3 Synthesis of N-CDs

The preparation of N-CD was based on a previous study (Tian et al., 2017). In brief, 1 g of citric acid and 2 g of urea were dissolved in 15 mL of deionized water using ultrasound, and then the solution was placed in a 25 mL Teflon reactor and heated at 160 °C for 4 h. The resulting aqueous solution of N-CDs was added to 20 mL of ethanol and centrifuged at high speed for 15 min. Finally, it was purified with a dialysis membrane in an ultrapure water (molecular weight cut-off 1000 Da) system for 24 h, followed by lyophilization for 72 h.

### 2.4 Synthesis of AuNCs

The AuNCs were prepared according to the method with a few emendations (He et al., 2018). At room temperature and under light protection, 5 mL of pre-prepared 10 mM HAuCl_4_ solution and 5 mL of BSA were added into a beaker, stirred for 30 min, then 500 μL of NaOH was added, and temperature was raised to 60 °C with continued vigorous stirring for 10 h in the dark. The resulting reaction solution was centrifuged at high speed for 10 min and further purified overnight in an ultrapure water (molecular weight cut-off 1000 Da) system using a dialysis membrane. The resulting brown solution of AuNCs was stored frozen at 4 °C protected from light.

### 2.5 Preparation of N-CDs/AuNCs@ZIF-8 nanocomposites

Briefly, 5 mL of 0.2 M Zn(NO_3_)_2_•6H_2_O methanolic solution was mixed with 20 mL of 0.2 M 2-methylimidazole methanolic solution in a 100 mL beaker, after which 6 mL of AuNCs and 200 μL of N-CDs solutions were added and stirred at 27 °C for 16 h in the absence of light. The mixed solvent was then centrifuged at high speed for 15 min, and the sediment was gathered and rinsed four times with methanol solution and vacuum cooled at 60 °C for 12 h to obtain the N-CDs/AuNCs@ZIF-8 powder.

ZIF-8 was prepared in the same way described above, except that AuNCs solution and N-CDs solution were not added. 5 mL of 0.2 M Zn(NO_3_)_2_•6H_2_O methanolic solution was mixed with 20 mL of 0.2 M 2-methylimidazole methanolic solution in a 100 mL beaker and stirred at 27 °C for 16 h in the absence of light. The mixed solvent was then centrifuged at high speed for 15 min, and the sediment was gathered and rinsed four times with methanol solution and vacuum cooled at 60 °C for 12 h to obtain the ZIF-8 powder.

### 2.6 Construction of the ratiometric fluorescent nanosensor for Gly

The structure and detection principle of the dual-emissive N-CDs/AuNCs@ZIF-8 ratio fluorescent sensor is shown in Scheme 1. For the actual determination, 50 μL of various concentrations of Gly solution and 50 μL of AChE (1.5 mU/mL) solution were placed in a 1 mL EP tube and incubated at 37 °C for 30 min, then 50 μL of ATch (1 mM) solution was added and incubated for another 30 min. Then, 50 μL of Cu^2+^ (1.25 μM) solution was added sequentially, 50 μL of N-CDs/AuNCs@ZIF-8 nanocomposite solution and 50 μL of PBS buffer solution were added sequentially and incubated at 37 °C for 5 min. Subsequently, the composite solution was added to a 350 μL quartz cuvette to record the fluorescence intensity (*I*) of the two emission peaks of the composite solution at wavelengths of 410 nm for N-CDs and 650 nm for AuNCs using a fluorescence spectrometer with an exciting wavelength of 360 nm. The *I*_650_/*I*_410_ ratio value was calculated for quantitation of Gly.

### 2.7 Selectivity of the sensing platform

The selective capability of the designed ratiometric sensor platform for Gly was evaluated through several parallel experiments and measurements of fluorescence reactions toward other commonly monitored pesticides (thiram, carbendazim, atrazine and metribuzin) with different structures, types, and uses from Gly. For this evaluation, 50 μL of a mixed solution containing Gly and four other interfering pesticides (100 ng/mL) was filled into a 1 mL EP tube, followed by the addition of 50 μL of AChE solution (1.5 mU/mL) for incubation for 30 min at 37 °C. Afterward, 50 μL of ATch (1 mM) solution was added and incubated for another 30 min. Finally, 50 μL of Cu^2+^ (1.5 μM), 50 μL of N-CDs/AuNCs@ZIF-8 probe and 50 μL of PBS buffer solutions were sequentially added to the above-mixed solution, incubated for 5 min to record the fluorescence intensities *I*_410_ and *I*_650_ under an exciting wavelength of 360 nm to calculated and *I*_650_/*I*_410_ values.

### 2.8 Application of the proposed ratiometric fluorescent sensors for Gly detection

Recovery experiments were performed to investigate the accuracy and reliability of the novel ratiometric fluorescence sensing platform in practical application through spiking the edible and medicinal malt samples with low (2 ng/mL), medium (50 ng/mL) and high (100 ng/mL) concentrations of Gly. They were subjected to ultrasonic treatment for extracting Gly with 5 mL of ultrapure water as extraction solvent, followed by sonicating for 20 min. The fortified malt sample solution was centrifuged at 6000 rpm at a low temperature for 15 min, and the supernatant was collected and filtered through a 0.22-μM filter membrane to remove matrix interferences. The filtrate was gathered to obtain the analytical solution for Gly determination using the developed ratiometric fluorescence sensing platform.

## 3 Results and discussion

### 3.1 Characterization of N-CDs

**Fig. 1:**
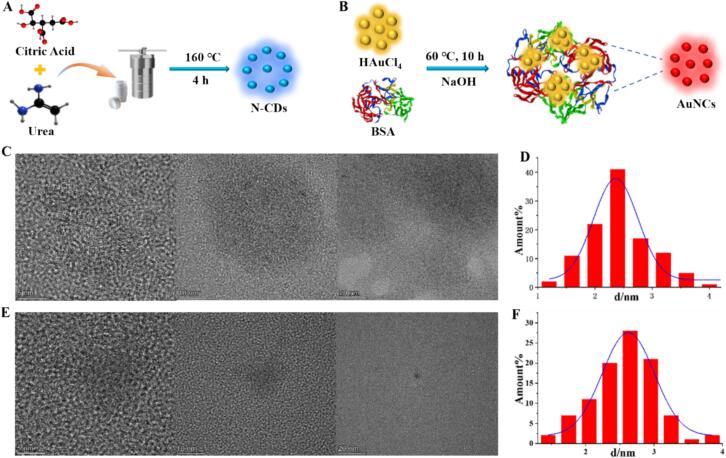
Synthesis of (A) N-CDs, and (B) AuNCs. (C) HR-TEM images, and (D) Particle size distribution of N-CDs. (E) HR-TEM images, and (F) Particle size distribution of AuNCs.

N-CDs were prepared by hydrothermal synthesis with citric acid and urea as reactants and heated at 180 °C for 4 h as shown in Fig. 1A, which presented blue emission under a UV light. HR-TEM characterization of these blue-emissive N-CDs was carried out and was illustrated in Fig. 1C, which showed that the N-CDs obtained were globular with uniform distribution in water. Its lattice stripe spacing was 0.21 nm, which was same to the description (Zhang & Guo, 2021). The particle size distribution in Fig. 1D showed that the synthesized N-CDs has a particle size distribution ranging from 2.2 to 2.6 nm with a mean size of 2.44 nm. The above results indicated that the synthesized N-CDs were spherical nanoparticles of homogeneous size with outstanding water solubility and dispersity without self-clustering.

### 3.2 Characterization of AuNCs

The synthesis of AuNCs with red fluorescence was achieved through the reaction of BSA and HAuCl_4_ as reducing and stabilizing agent, which was shown in Fig. 1B. From the HR-TEM characterization images of the red-emissive AuNCs in Fig. 1E, it could be found that the prepared AuNCs presented homogeneous and spherical structures in water solution with satisfactory dispersion and no aggregation. The particle size of AuNCs in Fig. 1F ranges from 2.6 to 2.8 nm, with a mean size of 2.45 nm.

### 3.3 Characterization of the N-CDs/AuNCs@ZIF-8 nanoprobes

**Fig. 2:**
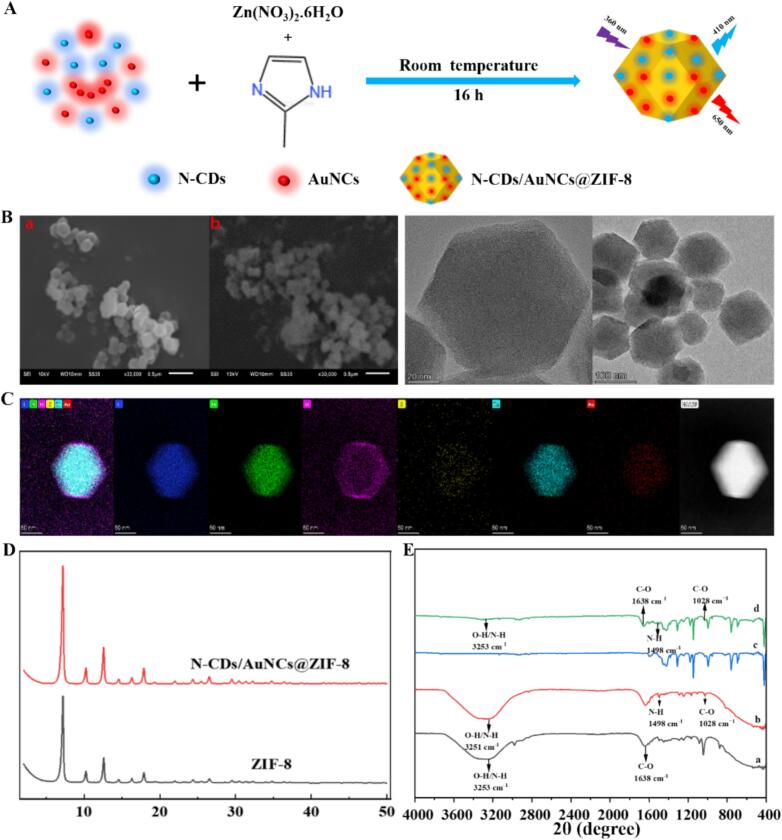
(A) Synthesis of N-CDs/AuNCs@ZIF-8. (B) SEM images of ZIF-8 (*a* on the left), SEM (*b* on the left) and TEM (on the right) images of N-CDs/AuNCs@ZIF-8. (C) HAADF-STEM images of N-CDs/AuNCs@ZIF-8 and the corresponding elemental mappings (C, N, O, Zn, Au, and S). (D) XRD spectra of ZIF-8 and CDs/AuNCs@ZIF-8. (E) FTIR spectra of (a) N-CDs, (b) AuNCs, (c) ZIF-8, and (d) N-CDs/AuNCs@ZIF-8.

The prepared N-CDs/AuNCs@ZIF-8 nanoprobes shown in Fig. 2A were first structurally characterized by the HR-TEM analysis. The two SEM images on the left in Fig. 2B showed that the synthesized N-CDs/AuNCs@ZIF-8 nanoprobe had a homogeneous morphology with a rhombic ortho dodecahedron. No remarkably changed morphology was observed in N-CDs/AuNCs@ZIF-8 in comparison with ZIF-8. The TEM images on the right in Fig. 2B show the successfully encapsulated N-CDs and AuNCs in ZIF-8.

The preparation of N-CDs/AuNCs@ZIF-8 nanocomposite was further validated by high-angle annular dark-field scanning transmission electron microscopy (HAADF-STEM) and associated element mapping analyses. Fig. 2C showed that the N, O, and Zn elements were clearly present alone in the nanocomposite, indicating the generation of the ZIF-8 backbone. In the meantime, Au, S and C components were uniformly dispersed in ZIF-8 molecular sieves, demonstrating the effective encapsulation of N-CDs and AuNCs nanoparticles. In addition, the XRD spectrum of N-CDs/AuNCs@ZIF-8 in Fig. 2D was consistent with that of pure ZIF-8 demonstrating that the encapsulation of N-CDs and AuNCs did not influence the crystal growth of ZIF-8. Then, FTIR spectra in Fig. 2E were performed to characterize the synthesis of the nanocomposite. The peak at 3253 cm^−1^ in Fig. 2E-a was ascribed to the stretching vibrations of the O—H and N—H bonds of the N-CDs, the peak at 1638 cm^−1^ was consistent with the vibration of the C—O bond. The FTIR spectrum of AuNCs in Fig. 2E-b confirmed the presence of O—H or N—H stretching vibrational peak at 3251 cm^−1^, and the peaks at 1498 cm^−1^ and 1028 cm^−1^ were attributed to the telescopic vibration peaks of N—H and C—O (Zhang et al., 2022). The absorption peaks at 3253 cm^−1^, 1635 cm^−1^, 1498 cm^−1^, and 1028 cm^−1^ of N-CDs and AuNCs were also observed in the spectrum of N-CDs/AuNCs@ZIF-8 nanocomposite in Fig. 2E-d, but not in the spectrum of pure ZIF-8 composite in Fig. 2E-c. All these findings indicate the success of the nanocomposite synthesis.

### 3.4 Sensing mechanism of the N-CDs/AuNCs@ZIF-8 ratiometric nanoprobes for Gly detection

The dual-emissive N-CDs/AuNCs@ZIF-8 ratiometric nanoprobes were obtained by encapsulating N-CDs and BSA-modified AuNCs into the ZIF-8 metal framework. In the addition of Cu^2+^, the red fluorescence of AuNCs was quenched and the blue fluorescence of N-CDs was stable. It has been reported that the fluorescence of AuNCs could be quenched by Cu^2+^ rather than Cu^+^ (Hada, Zetes, Focsan, Nagy-Simn, & Craciun, 2021) due to the coordination binding between AuNCs and Cu^2+^. Thus, the red fluorescence of AuNCs was quenched after adding Cu^2+^ to the sensing system. However, Tch, the product of ATch hydrolyzed by AChE, could also coordinate with Cu^2+^ *via* the sulfhydryl (-SH) group, inducing a marked red fluorescence recovery in the sensing system. While the introduced Gly could prevent the action of AChE, thereby preventing the generation of Tch and the red fluorescence quenching of AuNCs.

**Fig. 3:**
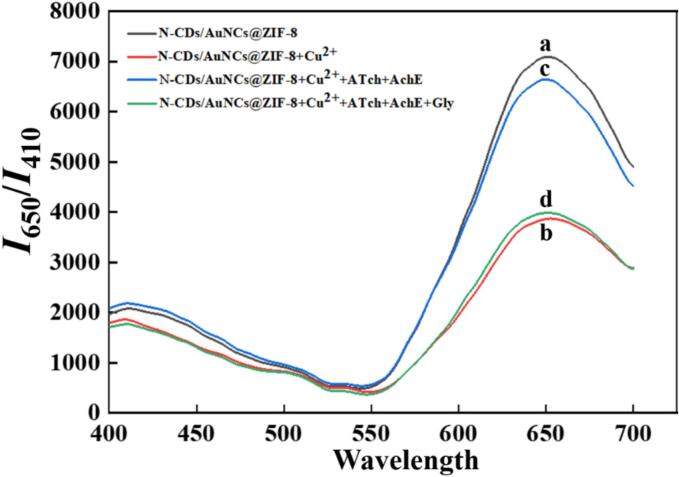
Fluorescence spectra of (a) N-CDs/AuNCs@ZIF-8, (b) N-CDs/AuNCs@ZIF-8 + Cu^2+^, (c) N-CDs/AuNCs@ZIF-8 + Cu^2+^+ATch+AChE, and (d) N-CDs/AuNCs@ZIF-8 + Cu^2+^+ATch+AChE+Gly.

This sensing mechanism was reflected in Fig. 3. The largest *I*_650_*/I*_410_ value was observed for the N-CDs/AuNCs@ZIF-8 nanoprobes (curve *a*), which reached the smallest when Cu^2+^ was added (curve *b*) due to the quenching effect of Cu^2+^ on the fluorescence of the nanoprobes, and then exhibited a remarkable increase after introducing ATch and AChE (curve *c*) that was a little smaller than that of the N-CDs/AuNCs@ZIF-8 nanoprobes owing to the binding of Tch with Cu^2+^ and the resultant fluorescence recovery, which was largely decreased when Gly was introduced (curve *d*) because of the inhibitory effect of Gly on the activity of AChE. Therefore, by integrating the ratiometric N-CDs/AuNCs@ZIF-8 fluoresence nanoprobes with Cu^2+^ and AChE, an ultrasensitive sensing platform was designed for Gly detection.

### 3.5 Optimization of sensing parameters

To achieve the best possible detection performance, key conditions such as Cu^2+^ addition concentration, solution pH, incubation time of Cu^2+^ with Gly and type of buffer solution were optimized.

**Fig. 4:**
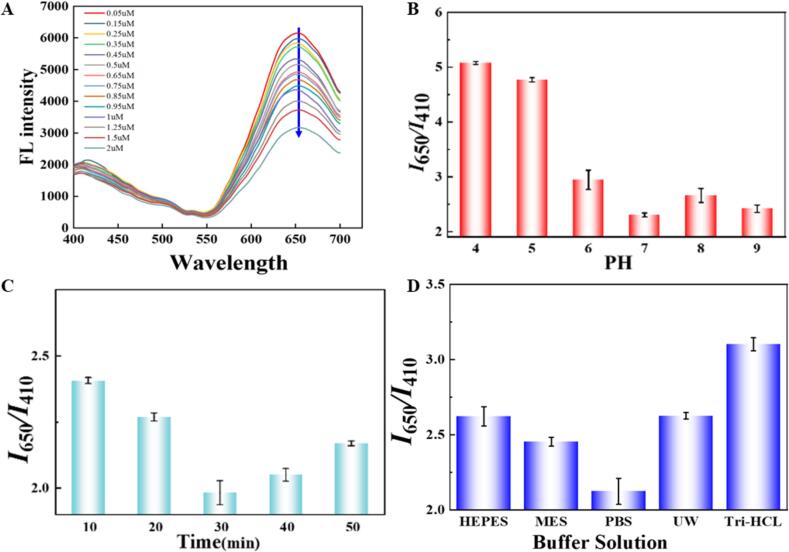
Condition optimization of the (A) added concentration of Cu^2+^, (B) solution pH, (C) incubation time between Cu^2+^ and Gly, and (D) type of buffer solution.

Different concentrations of Cu^2+^ induced diverse effects on the fluorescence responses of AuNCs, resulting in significant changes in the detected signal intensity ratio *I*_650_*/I*_410_. Therefore, 0.05–2 μM of Cu^2+^ was first considered to select the most suitable concentration. As shown in Fig. 4A, with increasing the concentrations of Cu^2+^ in the sensing system, the fluorescence intensity of AuNCs at 650 nm was reduced, and when the concentration reached 1.25 μM, approximately 80 % of the red fluorescence of AuNCs was quenched, while the blue fluorescence of N-CDs was almost unchanged. Therefore, 1.25 μM was chosen as the optimal concentration of Cu^2+^.

The solution pH of the ratiometric fluorescent sensing system affected the protonation and deprotonation processes of the fluorescent nanomaterials, and further influenced the fluorescence intensity of N-CDs and AuNCs, resulting in changes of *I*_650_*/I*_410_ value. Thus, the solution pH was adjusted by adding an acidic solution to obtain a suitable environment system for the best detection of Gly. By comparing the *I*_650_*/I*_410_ values of the sensing system at pH 4–9, it could be observed from Fig. 4B that the lowest *I*_650_*/I*_410_ value was obtained when the sensing system was in a neutral environment (pH 7). This might be due to the fact that the various molecules or ions in the sensing system are in a more stable state, which was conducive to the occurrence of specific chemical reactions or interactions allowing for optimal signals. Thus, the solution system was controlled at pH 7 for Gly detection in the subsequent experiments.

To ensure the completely identification of Gly, the incubation time of Gly with the N-CDs/AuNCs@ZIF-8 + Cu^2+^+ ATch + AChE probes was examined in 50 min with a 10-min interval. As shown in Fig. 4C, the *I*_650_/*I*_410_ values gave a decreasing to increasing trend within 50 min, and the lowest value was obtained at 30 min. Thus, the incubation time of Gly with the complete probes were set at 30 min.

The buffer solution maintains the pH of the sensing system during the sensing process to reduce the effect of pH on the sensing system. Here, the effects of five buffer solution systems including PBS, HEPES, Tris-HCl, MES, and ultrapure water (UW) on the performance of the sensing system were compared. Fig. 4D showed that the lowest *I*_650_*/I*_410_ value was achieved when using PBS solution. This might be beneficial of the specific ionic strength and pH stability of this buffer to provide a suitable chemical environment for the sensing system. The specific ionic composition might interact with molecules or ions in the sensing system for the good dispersion of N-CDs/AuNCs@ZIF-8 probs. Therefore, PBS buffer was chosen as the solution throughout the experiments.

### 3.6 Analytical performance assessment

**Fig. 5:**
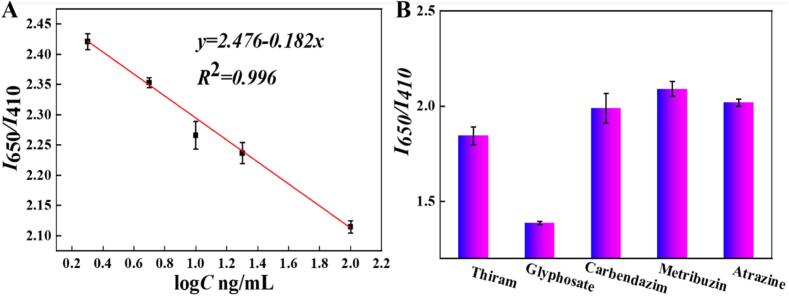
(A) Linear relationship between *I*_650_*/I*_410_ and the logarithm of Gly concentration; (B) Specificity evaluation of the N-CDs/AuNCs@ZIF-8 nanoprobes toward Gly and other four pesticides at 100 ng/mL.

In the ideal experimental circumstances, the sensing performance of the new dual-emissive N-CDs/AuNCs@ZIF-8 probes-assisted ratiometric fluorescent nanosensor for target Gly was investigated. Different concentrations of Gly (2–100 ng/mL) were added into the N-CDs/AuNCs@ZIF-8 + Cu^2+^+ ATch + AChE system for a 30 min incubation. Then, the Gly-induced fluorescence intensities *I*_650_ and *I*_410_ were recorded to calculate the *I*_650_*/I*_410_ values. A good linear relationship in Fig. 5 A was found between *I*_650_*/I*_410_ (*y*) values and the logarithms of Gly concentrations from 2 to 100 ng/mL with a linear calibration curve of *y* = 2.476–0.182*×* (*R*^2^ = 0.996). The limit of detection (LOD) of the proposed fluorescent nanosensor was calculated to be 1.92 ng/mL by the equation LOD = (3σ/k) (10 blanks were used to calculate σ).

In addition, Table 1 compares the performance of the newly developed ratiometric fluorescence nanosensor with various other Gly detection sensing platforms and reveals that the ratiometric fluorescence sensor developed in this study can simultaneously achieve low LOD and a desirable linear detection range, offering an ideal strategy for the detection of Gly residues in complex matrix samples.

**Table 1:** Comparison of the developed ratiometric fluorescent nanosensor with other sensing methods for Gly detection.

Sensing method	Material	Linear range	LOD	Refs.
Electrochemical sensor	MIP/MWCNTs-Au/GCE	1.73–400 ng/mL	0.24 ng/mL	(Su et al., 2023)
Colorimetric sensor	CDs-Au NCs	0–180 nM	4.19 nM (0.7 ng/mL)	(Zhang et al., 2022)
Electrochemical sensor	NH_2_-Bi-MOF	0.80–200 μmol/L	0.2 μmol/L (33.8 ng/mL)	(Wan, Pang, Yang, Yang, & Shen, 2023)
Fluorescent sensor	NH_2_-MIL-88(Fe)@RhB	0.6–15 μmol/L	0.18 μmol/L (30.4 ng/mL)	(Wan, Pang, Feng, & Shen, 2022)
Fluorescent sensor	DNA-AgNCs	15–100 ng/mL	5 ng/mL	(Yang et al., 2022)
Fluorescent sensor	N-CDs@PCN-222	0–8 mg/L	9.06 μg/L (9.06 ng/mL)	(Luo et al., 2022)
Ratiometric fluorescent sensor	CDs/AuNCs@ZIF-8	2–100 ng/mL	1.92 ng/mL	**This work**

### 3.7 Selectivity evaluation

To assess the specific detection ability of the developed ratiometric fluorescent nanosensor, its selectivity was evaluated by comparing the *I*_650_*/I*_410_ value induced by adding the target Gly and four other interfering pesticide molecules including thiram, carbendazim atrazine and metribuzin at 100 ng/mL. As could be seen in Fig. 5B, Gly exhibited the lowest *I*_650_*/I*_410_ value among the five pesticide molecules, which indicated the high selectivity of the newly developed N-CDs/AuNCs@ZIF-8 dual-emissive nanoprobes for Gly pesticide.

### 3.8 Malt sample analysis

To verify the accuracy and usability of the new-developed ratiometric fluorescence nanosensor, actual malt samples were chosen for the assay. Different concentrations of Gly were added to the actual blank (Gly-free) samples using the norm addition way and the spiked samples were processed as described above to determine recoveries. Table 2 showed that the mean recoveries of the three spiked samples ranged from 93.67 % to 108.2 % with RSD lower than 5.0 %, which were all in satisfactory requirements. These demonstrated the high accuracy and promising application ability of the proposed approach for the rapid, sensitive and precise detection of Gly in malt and other food samples.

**Table 2:** Determination of Gly in malt samples (*n* = 3).

Sample	Spiked (ng/mL)	Founded (mean ± SD) (ng/mL)	Recovery (%)	RSD (%)
1#	2	2.058 ± 0.06	102.9	3.5
2#	50	46.8 ± 1.70	93.67	4.4
3#	100	108.13 ± 2.02	108.2	2.3

## 4 Conclusion

In summary, a dual-emissive N-CDs/AuNCs@ZIF-8 nanoprobes based “signal on-off-on-off” ratiometric fluorescent nanosensor was fabricated to selectively recognize and precisely detect Gly. In this detection mechanism, the obtained N-CDs/AuNCs@ZIF-8 nanoprobes exhibited blue and red fluorescence responses (signal-on), but the red fluorescence of AuNCs were quenched by the added Cu^2+^ (signal-off). While, the coordination binding of Tch with Cu^2+^ would recover the fluorescence e of AuNCs (signal-on). Due to the inhibitory activity of target Gly on AChE activity to prevente the production of Tch, the red fluorescence of AuNCs was quenched again (signal-off). By calculating the ratio *I*_650_*/I*_410_ values of AuNCs to N-CDs, Gly in malt samples could realize accurate determination owing to the anti-interference ability from external environmental factors and the self-correction characteristics of the two emission signals. Under the optima conditions, the developed method could achieve Gly determination in a range of 2–100 ng/mL with good linearity, and the detection limit was 1.92 ng/mL. This study provided a reliable sensing strategy with simple preparation process of the ratiometric fluorescent probes, as well as merits of no heavy equipment, rapid preparation, large-scale production, simple sample treatment, easy operation, high response, good accuracy and practicability. In addition, the developed method displayed potential for the simultaneous determination of Cu^2+^ and Gly in real samples, broadening its application fields for food safety and environmental monitoring.

## CRediT authorship contribution statement

**Doudou Lei:** Writing – original draft, Formal analysis, Data curation. **Lingling Li:** Resources, Methodology. **Pengyue Song:** Methodology. **QingBin Xu:** Investigation, Formal analysis. **Lihua Huang:** Validation, Investigation. **Xiao Ma:** Investigation, Data curation. **Lidong Zhou:** Validation, Formal analysis. **Weijun Kong:** Writing – review & editing, Supervision, Funding acquisition, Conceptualization.

## Declaration of competing interest

The authors declare that they have no known competing financial interests or personal relationships that could have appeared to influence the work reported in this paper.

## Data Availability

Data will be made available on request.
