# Corrigendum to “Construction of a N-CDs/AuNCs@ZIF-8-assisted ratiometric fluorescent nanosensor for glyphosate detection in edible and medicinal malt” [Food Chem.: X 24 (2024) 101983]

**DOI:** 10.1016/j.fochx.2025.102503

**Published:** 2025-05-08

**Authors:** Doudou Lei, Lingling Li, Pengyue Song, QingBin Xu, Lihua Huang, Xiao Ma, Lidong Zhou, Weijun Kong

**Affiliations:** a State Key Laboratory of Southwestern Chinese Medicine Resources, Pharmacy College, Chengdu University of Traditional Chinese Medicine, Chengdu 611137, China; b Institute of Medicinal Plant Development, Chinese Academy of Medical Sciences and Peking Union Medical College, Beijing 100193, China; c School of Traditional Chinese Medicine, Capital Medical University, Beijing 100069, China

The authors regret the following errors:

1. **Figure Correction**
• Page 6, Fig. 3: The y-axis label “*I_650_/I_410_*” should be “FL intensity (a.u.)”
• **Text Correction**

Page 4, last paragraph, first line:

• Original text: “This sensing mechanism was reflected in Fig. 3. The largest *I_650_/I_410_* value was observed for the N-CDs/AuNCs@ZIF-8 nanoprobes (curve a)”.
• Corrected text: “This sensing mechanism was reflected in Fig. 3. The highest FL intensity was observed for the N-CDs/AuNCs@ZIF-8 nanoprobes (curve a)”.

**Rationale for Corrections:**

1. Standardization of fluorescence intensity terminology throughout the manuscript
2. Consistent use of conventional fluorescence reporting format
3. More accurate description of fluorescence signal characteristics

**Impact Statement:**

These corrections are purely terminological and do not affect:

• Any experimental data or results
• The methodology or conclusions of the study
• The scientific validity of the research

The authors would like to apologise for any inconvenience caused.

